# IoT Hierarchical Topology Strategy and Intelligentize Evaluation System of Diesel Engine in Complexity Environment

**DOI:** 10.3390/s18072224

**Published:** 2018-07-10

**Authors:** Jiangshan Liu, Ming Chen, Tangfeng Yang, Jie Wu

**Affiliations:** 1 School of Mechanical Engineering, Tongji University, Shanghai 201804, China; liujiangshan@tongji.edu.cn (J.L.); 1710020@tongji.edu.cn (J.W.); 2 Kunming Yunnei Power Co., Ltd., Kunming 650217, China; tangfeng_yang@163.com

**Keywords:** complexity environment, sensor network, topology strategy, AHP, intelligentize evaluation system

## Abstract

In complex discrete manufacturing environment, there used to be a poor network and an isolated information island in production line, which led to slow information feedback and low utilization ratio, hindering the construction of enterprise intelligence. To solve these problems, uncertain factors in the production process and demands of sensor network were analyzed; hierarchical topology design method and the deployment strategy of the complexity industrial internet of things were proposed; and a big data analysis model and a system security protection system based on the network were established. The weight of each evaluation index was calculated using analytic hierarchy process, which established the intelligentized evaluation system and model. An actual production scene was also selected to validate the feasibility of the method. A diesel engine production workshop and the enterprise MES were used as an example to establish a network topology. The intelligence level based on both subjective and objective factors were evaluated and analyzed considering both quantitative and qualitative aspects. Analysis results show that the network topology design method and the intelligentize evaluation system were feasible, could improve the intelligence level effectively, and the network framework was expansible.

## 1. Introduction

At present, the fields of aerospace, marine ships and automobiles have become the main industries to develop vigorously in a country, and diesel engine is one of the most important parts for power energies in these fields, thus is worthy of scientific research [1,2]. With the development of science and technology, the new manufacturing revolution represented by “German industrial 4.0” and “made in China 2025” are changing the mode of production of diesel engine [3,4]. The traditional small variety and mass production mode ignore the personalized customization demand. The original production workshop network of the diesel engine enterprise is poor, with the phenomenon of isolated information island being prominent [5], causing the bottom information to not be fed back in time, the production line being unable to respond to the change of production under the uncertain factors quickly, and ultimately the production efficiency and product quality being affected. Therefore, a network topology designed according to the production status and characteristics of the diesel engine enterprise can realize the interconnection and intercommunication between the production factors, and improve the intelligent production level of the enterprise [6,7].

As a special network, Industrial Internet of Things (IIoT) network is widely used in manufacturing industry, especially in the discrete industry. IIoT network is used to collect, analyze and apply the information from the state of production, raw materials, etc. [8]. The performance of the network is related to the topology design and the data transmission strategy [9,10]. However, there are some uncertainties in the complexity environment, so the topology strategy and design method of the IIoT network are strictly required. Designers are usually not concerned with the network structure, thus network performance decreased when there are many data, and could even shut down due to security problems, thus influence the intelligence level of an enterprise [11,12]. To solve this problem, our contribution is to further improve the IIoT network performance. We present a hierarchical topology design method and the deployment strategy of the complexity IIoT network, which concerns the uncertain factors and sensor network demands. The big data analysis model and system security protection system are established. Based on the network topology structure model, the AHP method is used to calculate the weight of each evaluation index and establish the intelligentize evaluation model.

The rest of this article is organized as follows. First, the research status of the network is introduced from several aspects, and the contributions and shortcomings of each researcher in the network topology are analyzed. Then, the present situation and existing problems in diesel engine enterprise is analyzed, and the uncertain factors in production process are determined. After that, the topology strategy of IIoT network and evaluation of intelligentized manufacturing is designed, including hierarchical structure of network, big data analysis model, security protection system based on network and evaluation model of intelligent evaluation index, which is analyzed by AHP method. Then, the IIoT network topology strategy in diesel engine enterprise is designed, including foundry workshop, machining workshop and workshop MES. Finally, a case is presented to analyze the intelligentized level of the enterprise. The results show that the method proposed in the article can comprehensively test and evaluate the level of enterprise intelligence.

## 2. Literature Review

Huang et al. [13,14] deployed the Radio Frequency Identification (RFID) on the equipment and materials of production line to store and transfer the production information in real time. Lin et al. [15] deployed sensor networks based on group to obtain production status and main information in real time; analyzed the process information; and overcame the expansibility and heterogeneity of sensor deployment in large-scale industrial field. Lin et al. [16] deployed sensors to monitor the multilevel production line and optimized the performance of the workshop network by cascading network topology. Y Liu et al. [17] proposed a novel Quorum time slot adaptive condensing (QTSAC)-based MAC protocol for achieving delay minimization and energy efficiency for the wireless sensor networks (WSNs), which decreased the network latency and the network latency. As the basic element of the IIoT in workshop, the collection of information and the deployment of the transmission equipment are essential. Therefore, the research of IIoT topology deployment is based on the production information collected.

In the design of IIoT architecture in intelligent production workshop, Chen et al. [18] proposed a collaborative sensing intelligence framework; combined collaborative intelligence and industrial sensing intelligence; and obtained intelligent and efficient industrial production/service. Boyes et al. [19] reviewed the meaning of IIoT; developed an analysis framework for IIoT, which can be used to enumerate and characterize IIoT devices when studying system architectures; and analyzed security threats and vulnerabilities. Bassi et al. [20] established the IIoT reference framework for hardware, software and services, and used a practical case to introduce the functions of the framework. Kiljander et al. [21] combined pervasive computing with IIoT to build a new semantic interoperability architecture and mapped the central components of the IIoT architecture to the general model. Li et al. [22] combined AI technology with IIoT, proposed an intelligent manufacturing architecture based on AI, and obtained the result of case analysis. For the expansibility of the network, Okafor et al. [23] proposed IIoT model based on extended cloud, which changed the traditional network services and routing strategies. Most research on the network framework considers the ontology function, or changes the network construction strategy, while there is less research on how to deal with the network topology of large and high dimensional data under a complex and changeable environment.

From the research on IIoT performance topology design and the problems of long delay in network transmission and unbalanced load between subnetworks, Li et al. [24,25] used Non-dominated Sorting Genetic Algorithm (NSGA-II) method to solve the multi-objective optimization problems and obtained Pareto frontiers, which improved the performance of network transmission. Shao et al. [26,27] used neural network algorithm to solve the multi-objective network topology optimization problem and obtained the solution effectively. Wang et al. [28,29] proposed a method to optimize the design of communication network topology that took the minimum network delay as objective, and the cost of network design and network connective reliability as constraints, and used GA to search the optimal solution of network topology. Most research on improving network performance is aimed at several optimization objectives in the production process, with certain ideal constraints or assumptions, while the uncertainty, complexity and security problems in the actual workshop environment are less researched, as they are difficult to realize in the actual deployment of the network.

Although some researchers studied network topology design of intelligent production workshop from different aspects, these studies are be completely applicable to the real production workshop in an uncertain environment, as the expansibility of the network was not high enough. Therefore, based on the above research, this paper analyzes the uncertain factors and the existing problems in the production process according to the characteristics and requirements of the diesel engine production process; puts forward the hierarchical topology and the deployment strategy of the IIoT network; and establishes many analysis models and security protection systems for the IIoT. The influence factors of enterprise intelligence level are analyzed hierarchically, and the enterprise intelligent evaluation model is established. Using the production workshop and Manufacturing Execution System (MES) of diesel engine as an example, the feasibility of the network topology design strategy and the feasibility of the workshop intelligent evaluation system and the index weight calculation method are verified.

## 3. Problem Analysis

### 3.1. Present Situation and Existing Problems in Diesel Engine Enterprise

The diesel engine enterprise has mechanical workshop and foundry workshop. The mechanical workshop has many production lines, such as core making, melting and teeming. The foundry workshop has cylinder head, cylinder body and lower body lines. Each production line has M processes, and each process has N workplaces. There are many kinds of products involved in the same lines, which lead to many uncertain factors. When the foundation of the network is poor, it is difficult to respond to the change of production plan caused by uncertain factors in time. The main production status and existing problems of diesel engine enterprises are shown in Table 1.

### 3.2. Analysis of Uncertain Factors in Production Process

Diesel engine has many different complex parts. There are many uncertain factors in the internal and external environment of the production system, which influence the production process and makes the production resources and information impossible to be fully shared, so the production efficiency is low and can even cause shutdown phenomenon [30]. According to the characteristics and key influencing factors of the actual production workshop, the IIoT model and deployment strategy are constructed to realize networking control, interconnection, and intercommunication between factors of production, so it can respond to the plan change caused by the uncertain factors in time.

## 4. The Topology Design Strategy of IIoT Network and Evaluation Model of Intelligentize Manufacturing

### 4.1. The Topology Design Strategy of IIoT Network

**Figure 1:**
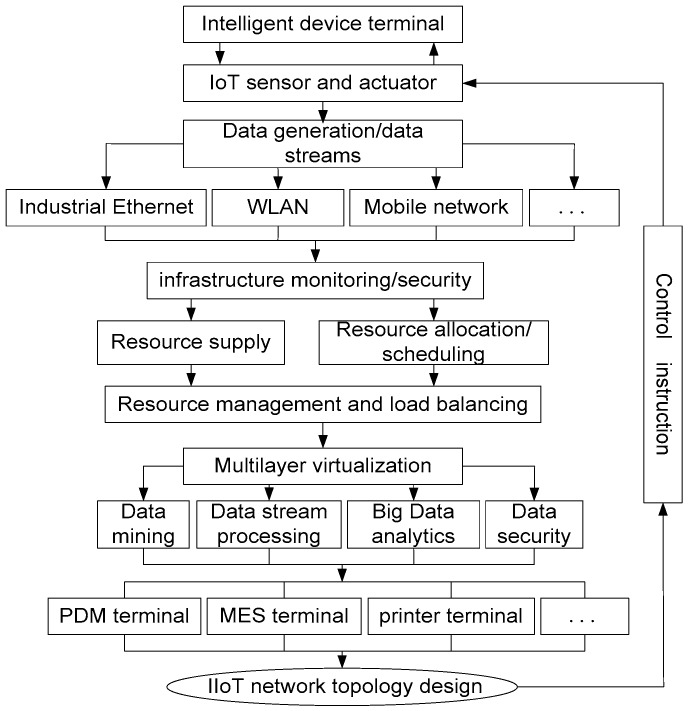
IIoT network design strategy process.

To better guide the IIoT network construction and let the enterprise realize intelligent transformation, after analyzing the characteristics of diesel engine production process and the uncertain factors in the production system, we can obtain the IIoT network topology design process shown in Figure 1.

**Figure 2:**
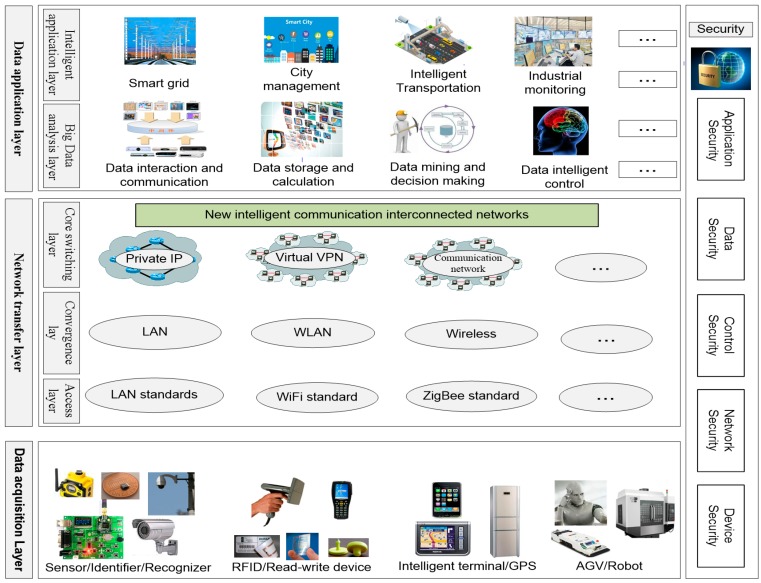
Hierarchical structure of IoT network.

When designing the network according to the above IIoT design strategy process, we need to consider the structural characteristics and functional requirements of the network itself. In general, the IoT network has three layers: data acquisition layer, network transfer layer and data application layer [31] (Figure 2). In the actual network topology design, we need to deploy it according to the characteristics of each layer.

In Figure 2, the main function the of data acquisition layer is to identify objects collect data; it includes sensors, RFID, Quick Response (QR) code, ZigBee, Bluetooth, etc. The network transfer layer includes mobile communication network, wireless sensor network, etc. It is used to deeply integrate network and transfer the data to the whole network. The data application layer can build an intelligent functional application platform for business requirements, and provide solutions for different users after analyzing and processing data, such as using cloud computing, artificial intelligence, large data analysis and other technologies. There are different security problems in the hierarchical structure of IoT network, which we group into five aspects, according to the basic principles of deep security defense: application security, data security, control security, network security and device security [32].

**Figure 3:**
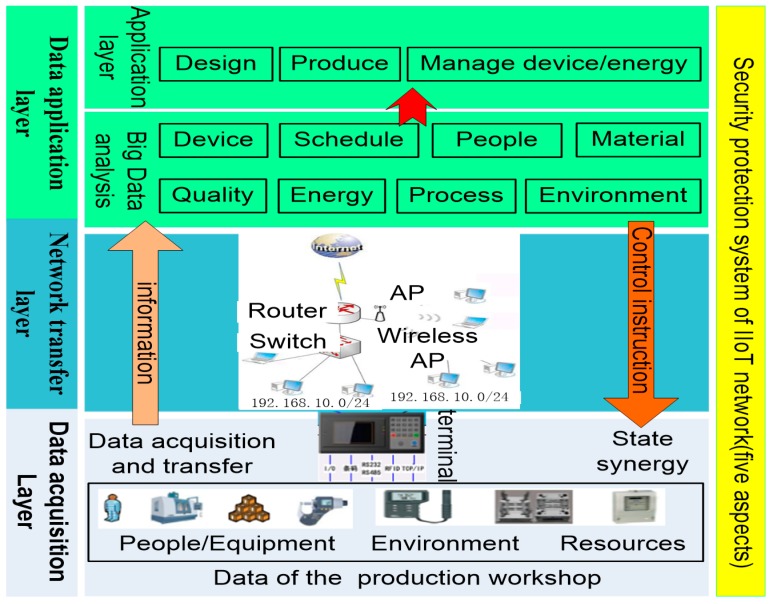
Hierarchical structure of IIoT network.

Based on the above method and structure, the IIoT network hierarchical structure is designed (Figure 3). An IoT network designed for production site needs to consider complex and changeable production environments. The network has particularity and expansibility and requires higher data analysis and system security.

In Figure 3, the data acquisition layer is used to identify and collect the state and data from people, equipment and raw materials. The data are transmitted to the data application layer through the network equipment, such as router, switches and other network devices in the network transfer layer. At the same time, the control instructions from the data application layer are also sent to the bottom of the network to control the production factors.

According to the characteristics of IIoT network, data application layer can be subdivided into big data analysis layer and intelligent application layer. Many complex data can be analyzed and processed with big data and cloud computing technology, which can be used to manage and control the equipment, materials and production process. The intelligent application layer can be applied to MES, Enterprise Resource Planning (ERP), Process Control System (PCS), etc., and used to schedule and diagnose the production resource, etc.

### 4.2. The Big Data Analysis Model and Security Protection System Oriented IIoT Network

**Figure 4:**
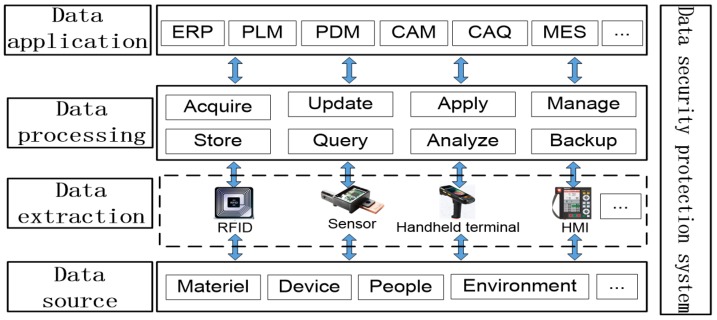
Big data analysis model in network structure.

As mentioned above, the network nodes and hierarchy structure of IIoT network have their particularity in the intelligent production mode. The analysis ability and real-time transmission requirement of data in the production process make it complicated [33]. The nodes of IIoT network include Programmable Logic Controller (PLC), sensor, actuator, etc. The transmission medium includes wired and wireless transmission lines. Most data produced and collected from intelligent devices are unstructured, and the intelligent production network system needs to have the ability to deal with data heterogeneity in real-time. Therefore, it is necessary to build a big data analysis model for network structure. As shown in Figure 4, the model includes acquisition, storage, analysis and application of data. The processed data can be used for real-time analysis, monitoring, and visualization of main production processes. It not only provides basic services for intelligent analysis, reasoning and decision making, but also reduces inventories, optimizes supply chains, and meets the personalized customization needs.

**Figure 5:**
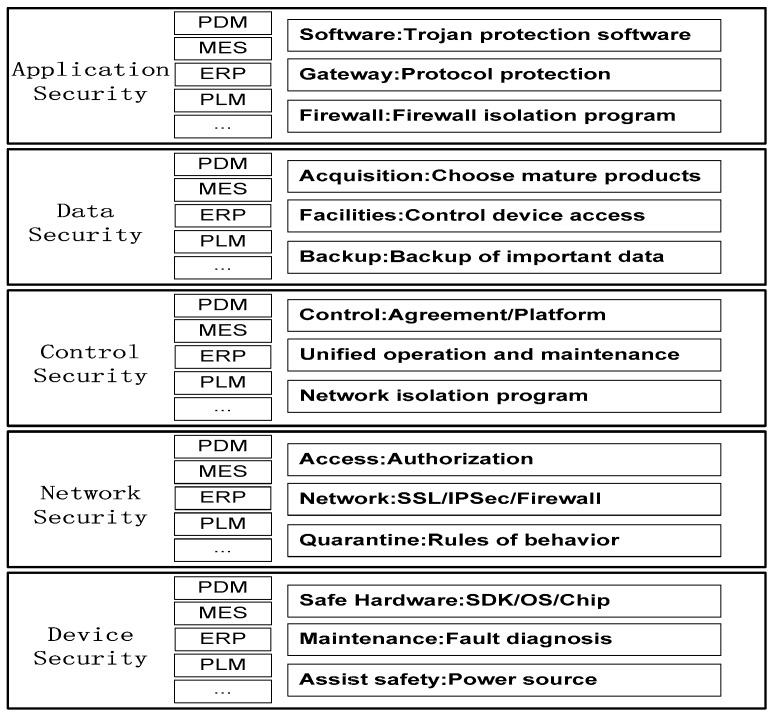
Safety protection system of production.

With the development of the production control system in the direction of intelligence, network and complexity, there are many kinds of production factors and complex data in the system, as well as many interconnected relationships among them; the complexity environment could easily cause security problems in the system [34]. Therefore, it is urgent to establish a perfect safety protection system to provide a guarantee for the safe operation of the production system. The security problems of the production system are mainly from the physical layer and the network layer. The physical layer includes the reliability of the sensors, actuators and controllers in the production system. The network layer includes data packet loss, delay, error code and leakage. Therefore, the topology design of the IIoT network needs to be able to prevent the system from being destroyed, minimize the damage and rapidly recover. The security protection system (Figure 5) is established according to the characteristics of the production system and the IoT solution components running in the various layers of the network. It can be analyzed from the aspects of device security, network security, control security, application security and data security. Safety measures are taken to ensure the safe operation of the production system.

### 4.3. Weight Coefficient and Evaluation Model of Intelligent Evaluation Index Based on IIoT Network

**Figure 6:**
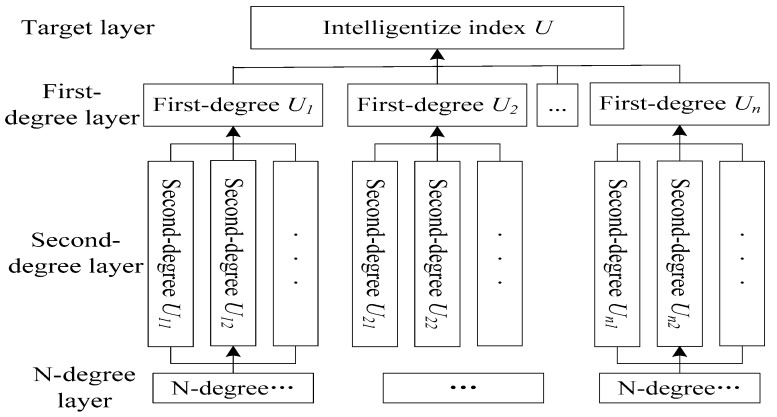
Hierarchical structure of intelligent evaluation index.

An intelligent evaluation model needs to be built to evaluate the degree of intelligence after establishing IIoT network topology and improving the intelligent level of the enterprise. Different industries and production types have different intelligent constraints that can be used Analytic Hierarchy Process (AHP) method divides an index into several layers to determine the weight coefficient of the intelligent evaluation index, which is quantitative and qualitative, as shown in Figure 6. Target evaluation layer is a comprehensive index quality of the intelligentize level, *U* = {*U*_1_,*U*_2_,...,*U_n_*}; *U_i_* is the *i-*th index in the first-degree evaluation index layer of target layer *U*, *U_i_* = {*U_i_*_1_,*U_i_*_2_,...,*U_im_*}, *i* ∈ (1,n); and *U_ij_* is the *j-*th index in the second-degree evaluation index layer of the first-degree index layer *U_i_*, *j* ∈ (1,m).

The judgment matrix of each evaluation index layer can be obtained after the evaluation according to hierarchical structure shown in Figure 6. The matrix form is *A* = *(a_ij_)_n__×__n_*, *i* = [1,*n*], *j* = [1,*n*], which is a positive reciprocal matrix, *a_ij_* = 1*/a_ji_*, as shown in Equation (1). The weight coefficient of each evaluation index can be calculated using Equation (1).

(1) $$A=\left[\begin{array}{cccc} 1 & a_{12} & \cdots & a_{1n}\\ a_{21} & 1 & \cdots & a_{2n}\\ \vdots & \vdots & \ddots & \vdots \\ a_{n1} & a_{n2} & \cdots & 1 \end{array}\right],$$

In Equation (1), the order of the judgment matrix *A* is the same as the number of evaluation indices. The value of *a_ij_* reflects the importance of each evaluation index, which generally uses a 1–9 scale. For odd numbers, *a_ij_* = 1 means that the element *i* has the same importance as element *j* on the higher level factors; the greater the value is, the more important the element is. For even numbers, *a_ij_* = 2 means that the importance of element *i* and element *j* is more than scale value 1, but less than value 2, and so on, for scale values 4, 6, and 8.

The intelligent evaluation index is divided into quantitative index and qualitative index. For the quantitative index, the maximum and minimum values are fixed values that need to be determined first; the maximum value *x_imax_* is the best value of the evaluation index after intelligent manufacturing, while the minimum value *x_imin_* is the value of the evaluation index when the intelligent manufacturing has not started. After determining the range of its value, dimensionless treatment is carried out according to Equation (2).

(2) $$x_{i}^{\prime }=\frac{x_{i}-x_{imin}}{x_{imax}-x_{imin}}\times 100,$$

To further explain the meaning of Equation (2), we use the *i*-th evaluation index as an example. First, the *i-*th evaluation index has its own initial value *x_i_*. After several intelligent evaluations, the *i-*th evaluation index is a range value because of the different opinions, *x_imax_* is the maximum value of the *i-*th evaluation index in the range value, *x_imax_* reflects the highest degree of intelligence, *x_imin_* is the minimum value of the *i-*th evaluation index in the range value, *x_imin_* reflects the lowest degree of intelligence, and $x_{i}^{\prime }$ is the dimensionless value of the *i-*th evaluation index, and 0 ≤ $x_{i}^{\prime }$ ≤ 100.

For the qualitative index of the evaluation index, subjective evaluation method can be used for analysis. Dimensionless treatment is carried out according to Equation (3).

(3) $$y_{j}^{\prime }=\frac{\sum_{k=1}^{n}x_{jk}-x_{jmin}-x_{jmax}}{n-2}\;,$$

In Equation (3), $y_{j}^{\prime }$ is the dimensionless value of the *j-*th evaluation index, *n* is the number of subjective evaluation, *y_jk_* is the *k-*th subjective evaluation initial value of the *j-*th evaluation index, *x_jmax_* is the maximum subjective evaluation value of the *j-*th index, *x_jmin_* is the minimum subjective evaluation value of the *j-*th index, and 0 ≤ $y_{j}^{\prime }$ ≤ 100.

Each evaluation index is interrelated, especially between the upper and lower index. The single weighting method needs to determine the single weight and then make a comprehensive evaluation, so it has some shortcomings, such as one-sidedness, boundedness, strong human factor, and it is easy for some important indices to be ignored. To overcome these shortcomings, we choose to use the weighted average model, which has rationality and scientificity, so the evaluation result is more objective, perfect and practical. The weighted average model is established in the form of Equation (4) after dimensionless processing of the intelligent evaluation index.

(4) $$H=\sum_{i=1}^{m}\sum_{j=1}^{n}\mu_{i}\delta_{j}x_{j}\;,$$

In Equation (4), *H* is an intelligent evaluation level of an enterprise, *μ_i_* is the *i-*th first-degree index weight coefficient value, *δ_j_* is the *j-th* second-degree weight coefficient value, and *x_j_* is the *j-*th evaluation index value, *i* ∈ (1,*m*)*, j* ∈ (1,*n*).

## 5. Application of the IIoT Network Topology Design Strategy in Diesel Engine Enterprise Intelligent Manufacturing

In Section 4.1, the IIoT network topology design strategy of the enterprise can realize the interconnection of the workshop from the bottom layer to the application layer, and improve the reliability and expansibility of the workshop. However, the feasibility of the diesel engine needs further verification. Therefore, the typical diesel engine production workshop and the enterprise MES are selected as an example for analysis.

### 5.1. IIoT Network Topology Design for the Foundry Workshop of Diesel Engine

**Figure 7:**
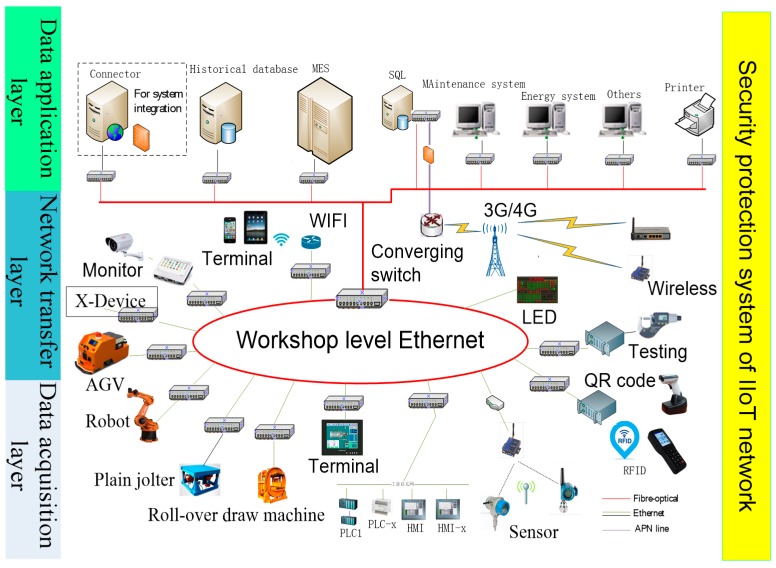
IIoT network topology of molding making production line.

The foundry workshop of diesel engine consists of mold making production line, core making production line, etc. Mold making production line involves many complex processes and big data. The IIoT network topology of mold making production line is established using the network topology design strategy described in the above discription, as shown in Figure 7.

Data acquisition layer is responsible for data acquisition and reception in the diesel engine modeling line, and includes AGV car, robot, plain jolter, roll-over draw machine, sensor, QR code scanner, etc. The network transfer layer includes wired network, wireless WiFi, etc. It is responsible for transferring data to the application layer to use for data analysis and application. The data application layer can analyze and process the data from the bottom production line, and then use it to monitor the equipment. It can meet the requirements for scheduling and remote maintenance, reduce machine downtime and improve productivity while extending current manufacturing resources.

**Figure 8:**
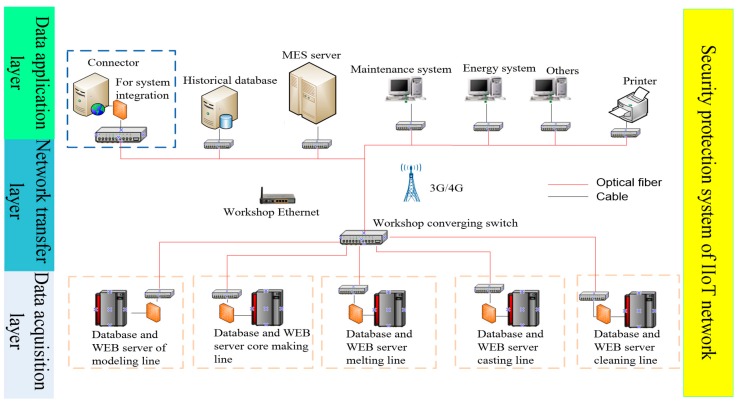
IIoT network topology of foundry workshop.

According to the IIoT network topology design strategy described above, a higher level network topology design structure is further realized, as shown in Figure 8.

The data layer of the sub network of each production line in the foundry workshop can get data from the intelligent unit. These data are transferred to the upper application platform through the Ethernet network and wireless sensor network of the network transfer layer. After analysis and processing, the data can serve the upper management system, such as the production management, energy management and remote fault diagnosis. They can also help control commands to control the bottom intelligent terminals.

### 5.2. IIoT Network Topology Design for the Machining Workshop of Diesel Engine

**Figure 9:**
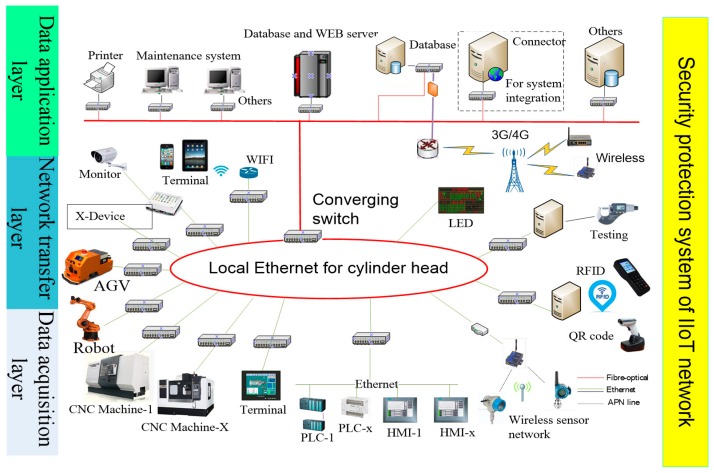
IIoT network topology of cylinder head production line.

To verify the general use of the network topology design method in this paper, another representative production workshop is chosen for further analysis. It can improve the intelligence level of the whole enterprise and facilitate the development of intelligent evaluation work. The machining workshop is composed of cylinder head production line, cylinder block production line, assembly lines, etc. The network topology of cylinder head production line is shown in Figure 9.

The data acquisition layer is composed of CNC machines, robots, operation terminals, PLC, RFID, etc. The network transfer layer is responsible for sending data to the application layer, including wired network, wireless network, etc. The data can be used for control and service of production line after data analysis and processing.

**Figure 10:**
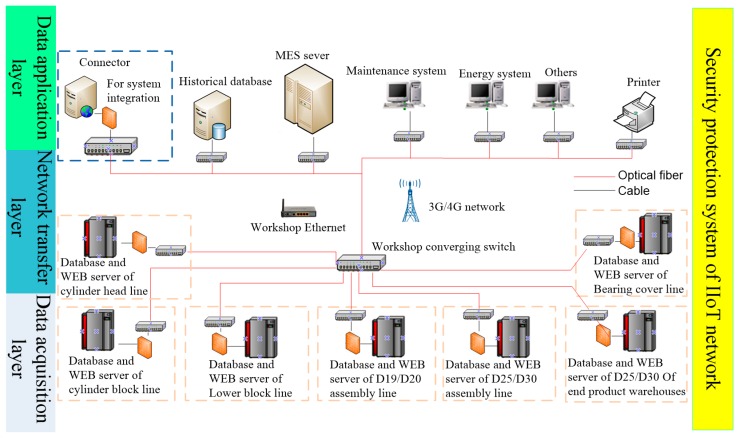
IIoT network topology of cylinder head production line.

Each production line of machining workshop has a separate management system; the subnetwork systems are connected to form the whole workshop network system, as shown in Figure 10. The data collected in the converging switch are obtained from each subnetwork of production line, and they are transferred through the network transfer layer to the application platforms. The data after analysis and processing can be used for the management and control of the workshop.

### 5.3. IIoT Network Topology Design of the Workshop MES in Enterprise

There are many complex data produced in the diesel engine production process [35]. The network connecting the production units of diesel workshop not only needs to meet the access of all kinds of equipment layer and control layer system, but also needs to meet the integrated access the upper information system, such as ERP, PLM, etc.

**Figure 11:**
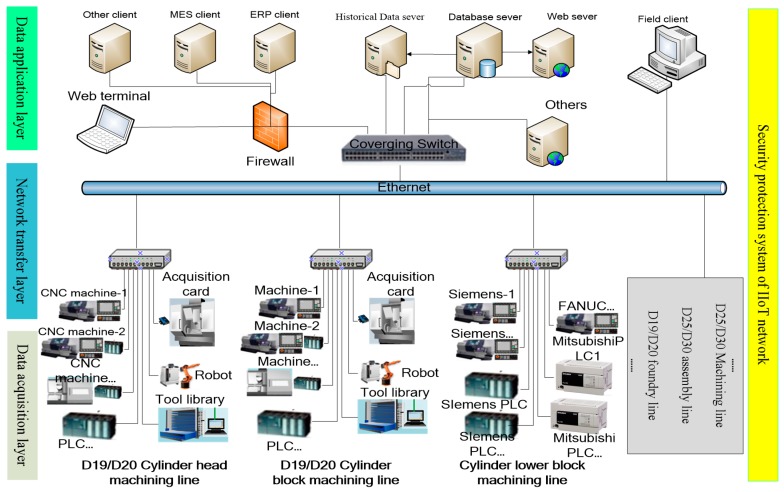
IIoT network topology design of workshop MES.

The MES of the diesel engine enterprise is used to control the production line, and the client can access data through the web server. To ensure the information security of the production process, the program and tool management of numerical control equipment is responsible for the special server. The office network computer and the MES/ERP server can obtain the data, which need to be isolated by a firewall. The computer can query the system state through the IE browser (Figure 11).

## 6. A Case of Analysis on Intelligent Evaluation of Diesel Engine Enterprise

### 6.1. The Establishment of Hierarchical Structure Model of Evaluation System

After finishing the network topology design of the production workshop and MES, the intelligentized level of the enterprise can be improved and the intelligence evaluation can provide evidence for the intelligent upgrading of the enterprise.

In this paper, some evaluation indices need subjective qualitative evaluation to determine the relative importance of each index. According to the intelligence evaluation system in Figure 6, the characteristics of the workshop can set up an enterprise intelligence hierarchical structure model [36], as shown in Table 2.

### 6.2. Result Analysis

After intelligence evaluation of the enterprise according to each index layer in Table 2, we can get the judgment matrix of the intelligent evaluation index in each layer of diesel engine workshop from Equation (1); solve the maximum eigenvalues and eigenvectors of judgment matrix of each layer; normalize the eigenvector; and then obtain weight vector between the evaluation indices of each layer, as shown in Table 3.

To get the weight coefficient of each layer of intelligent evaluation index, it is necessary to check the consistency of each judgment matrix. In addition to the two-order matrix *A_23_*, the CI (Consistency Index), the CR (Consistency Ratio) and RI (Random Consistency Index) of the remaining matrices are shown in Table 4.

In Table 4, the CR of each judgment matrix is less than 0.10, and we can think that the consistency of each judgment matrix is acceptable. Therefore, the weight coefficient of each intelligent evaluation index layer in Table 2 can be calculated, as shown in Table 5.

After the dimensionless processing of each intelligent evaluation index according to Equations (2) and (3), the weighted average (Equation (4)) is used to calculate the intelligent evaluation score, *H* = 92.66.

It can be divided into four levels according to the enterprise intelligent development index: entry level, primary level, intermediate level and advanced level [37].

(i) 86 ≤ *H* < 100, advanced intelligentized enterprise
(ii) 61 ≤ *H* < 85, intermediate intelligentized enterprise
(iii) 41 ≤ *H* ≤ 60, Primary intelligentized enterprise
(iv) *H* ≤ 40, entry intelligentized enterprise (single intelligent application).

According to the evaluation score, the network topology deployment strategy proposed in this paper can make every production unit in the workshop interconnected, effectively deal with the information island problem between the intelligent equipment in production, solve the problem of information sharing difficulty, improve the real time of production data, and make the production link of the enterprise possess better adaptability and flexibility. At the same time, the AHP method proposed in this paper can comprehensively test and evaluate the level of enterprise intelligence.

## 7. Conclusions

In uncertain production environment, it is always a key and difficult point to design a proper topology for multi-varieties and small-batch production tasks, and IIoT network in the workshop is always used to solve the problem of isolated information island in the production process. To solve these problems effectively, the production workshop and MES of diesel engine were used as the research object. A network topology design strategy and evaluation system was formed, improving the level of enterprise intelligence.

(1) According to production status, production demands and network characteristics, the topological process and deployment strategy of the IIoT were designed. The network topology design is expansible.
(2) The AHP method was used to establish the intelligent evaluation system for the diesel engine enterprise and the judgment matrix for each evaluation index layer was set up to verify the consistency of them. Finally, the weight coefficients between the evaluation indices of each layer were obtained. The weighted average model of the evaluation system was established to obtain the result of the intelligent evaluation level of the diesel engine enterprise.

The network topology design strategy and intelligent evaluation system established in this paper can be further extended to other applications. It has wide applicability for improving the intelligence level of enterprise. However, with the development of technology, people have different understanding and applications of intelligence, thus we need to conduct further research on different practices so that it has more positive effects in the future.

## Figures and Tables

**Table 1:** The production status and existing problems.

Name	Status	Problem
Equipment	Low intelligence, poor network	Information isolated island
Product	Multiple Species and Small Batch	Difficult to produce and manage
Design	Complex shape and high requirement	Difficult to Synergetic design
Schedule	Frequent change plan	Non-dynamic scheduling
Production	Complex data	Unable to share information
Management	No integration system	No interoperability

**Table 2:** Hierarchical structure model of enterprise intelligence evaluation.

Target Layer	First-Degree Index	Second-Degree Index
Intelligent evaluation index *U*	Decision support *U*_1_	Dynamic scheduling *U*_11_
		Supply chain management *U*_12_
		Order tracking *U*_13_
		Quality traceability *U*_14_
		Decision support *U*_15_
	Systems engineering *U*_2_	Data definition *U*_21_
		Data management *U*_22_
		Model Transfer *U*_23_
	System integration *U*_3_	MES and ERP integration *U*_31_
		ERP and PDM integration *U*_32_
	Economic benefit *U*_4_	Production cost *U*_41_
		Production efficiency *U*_42_
		Rejection rate *U*_43_

**Table 3:** Weight vector of each evaluation index judgment matrix.

Judgment Matrix	Maximum Eigenvalue	Eigenvector	Weight Vector
*A*_1_	5.15	(0.33,0.24,0.71,0.56)	(0.18,0.13,0.39,0.30)
*A*_21_	5.17	(0.41,0.18,0.26,0.85,0.09)	(0.23,0.10,0.15,0.47,0.05)
*A*_22_	3.02	(0.20,0.35,0.92)	(0.14,0.24,0.62)
*A*_23_	2	(0.95,0.32)	(0.75,0.25)
*A*_24_	3.11	(0.22,0.32,0.92)	(0.15,0.22,0.63)

**Table 4:** Consistency test of judgment matrix in each evaluation indices layer.

Judgment Matrix	CI	RI	CR
*A*_1_	0.0375	0.58	0.0646
*A*_21_	0.0425	1.12	0.0379
*A*_22_	0.0100	0.58	0.0172
*A*_24_	0.0550	0.58	0.0948

**Table 5:** Consistency test of judgment matrix in each evaluation indices layer.

First-Degree Index	Weight Coefficient	Second-Degree Index	Weight Coefficient
*U*_1_	0.18	*U*_11_	0.23
		*U*_12_	0.10
		*U*_13_	0.15
		*U*_14_	0.47
		*U*_15_	0.05
*U*_2_	0.13	*U*_21_	0.14
		*U*_22_	0.24
		*U*_2__3_	0.62
*U*_3_	0.39	*U*_31_	0.75
		*U*_32_	0.25
*U*_4_	0.30	*U*_41_	0.15
		*U*_42_	0.22
		*U*_43_	0.63
